# The role of diffusion magnetic resonance imaging in Parkinson's disease and in the differential diagnosis with atypical parkinsonism

**DOI:** 10.1590/0100-3984.2016-0073

**Published:** 2017

**Authors:** Romulo Varella de Oliveira, João Santos Pereira

**Affiliations:** 1 Full Member of the Colégio Brasileiro de Radiologia e Diagnóstico por Imagem (CBR), Masters Student in the Graduate Program in Medical Sciences at the Faculdade de Ciências Médicas da Universidade do Estado do Rio de Janeiro (FCM-UERJ), MD, Radiologist at the Hospital Universitário Pedro Ernesto (HUPE) and at the Clínica Alta Excelência Diagnóstica (DASA), Rio de Janeiro, RJ, Brazil.; 2 PhD, Full Member of the Academia Brasileira de Neurologia (ABN), Associate Professor, Coordinator of the Movement Disorders Sector of the Neurology Department of the Hospital Universitário Pedro Ernesto da Universidade do Estado do Rio de Janeiro (HUPE-UERJ), Rio de Janeiro, RJ, Brazil.

**Keywords:** Parkinson disease, Parkinsonian disorders, Supranuclear palsy, progressive, Multiple system atrophy, Neurodegenerative diseases/physiopathology, Diffusion magnetic resonance imaging.

## Abstract

Parkinson's disease is one of the most common neurodegenerative diseases. Clinically, it is characterized by motor symptoms. Parkinson's disease should be differentiated from atypical parkinsonism conditions. Conventional magnetic resonance imaging is the primary imaging method employed in order to facilitate the differential diagnosis, and its role has grown after the development of advanced techniques such as diffusion-weighted imaging. The purpose of this article was to review the role of magnetic resonance imaging in Parkinson's disease and in the differential diagnosis with atypical parkinsonism, emphasizing the diffusion technique.

## Abstract (pt)

A doença de Parkinson é uma das doenças neurodegenerativas mais comuns. Clinicamente, é caracterizada por sintomas motores, devendo ser diferenciada com outras doenças que cursam com parkinsonismo, definidas como parkinsonismo atípico. A ressonância magnética convencional é o principal método de imagem para auxiliar este diagnóstico diferencial e, atualmente, seu papel tem crescido com as técnicas avançadas, como a difusão. O objetivo deste artigo é revisar o papel da ressonância magnética na doença de Parkinson e no diagnóstico diferencial com parkinsonismo atípico, com ênfase na técnica de difusão.

## INTRODUCTION

### Parkinson's disease

Parkinson's disease is one of the most common neurodegenerative diseases, and its incidence increases progressively with age^(1)^. The estimated incidence of Parkinson's disease is 17.4 per 100,000 person-years in the 50- to 59-year age group, compared with 93.1 per 100,000 person-years in the 70- to 79-year age group, and the lifetime risk of developing the disease is 1-5%^(2,3)^. A population-based study conducted in Brazil showed that the prevalence of Parkinson's disease was 3.3% in the population over 64 years of age^(4)^. Due to the aging of Western populations, it is believed that the prevalence of the disease is on the rise.

First described by James Parkinson in 1817, Parkinson's disease occurs due to progressive loss of dopaminergic cells from the substantia nigra pars compacta, as well as α-synuclein aggregation in specific areas of the brainstem, spinal cord, and cortical regions^(1)^. Clinically, Parkinson's disease is characterized by motor symptoms such as bradykinesia, stiffness, resting tremor, and postural instability.

### Atypical parkinsonism

There are many disorders that can provoke symptoms characteristic of Parkinson's disease, although with atypical findings, and such disorders are therefore typically referred to, collectively, as atypical parkinsonism^(5)^. Despite having multiple causes, the term atypical parkinsonism is typically applied to the three most common sporadic neurodegenerative syndromes: multiple system atrophy, progressive supranuclear palsy, and corticobasal degeneration^(5)^. Atypical parkinsonism syndromes present distinct clinical characteristics, and, despite ongoing research, their etiopathogenesis remains unknown; to date, there are no known biomarkers of the syndromes and no specific treatments have been made available^(5)^.

In some cases, it can be difficult to make the differential diagnosis between Parkinson's disease and atypical parkinsonism, especially in the early stages of the disease. Pathological studies have shown that accuracy in the diagnosis of Parkinson's disease can be as low as 73.8%^(6)^. Making an accurate diagnosis is essential to the evaluation and prognosis, as well as to the decisionmaking process regarding the establishment of the best pharmacological and rehabilitative practices in patients with Parkinson's disease. Upon clinical examination, the presence of distinct neurological signs, such as ataxia and other cerebellar alterations, early autonomic dysfunction, dystonia, and vertical gaze paralysis, can suggest atypical parkinsonism.

The diagnosis of Parkinson's disease and atypical parkinsonism is essentially based on clinical findings, although imaging studies are important for making the differential diagnosis and for identifying structural lesions, such as vascular lesions and tumors.

Magnetic resonance imaging (MRI) is the main imaging method used in making the differential diagnosis between Parkinson's disease and atypical parkinsonism. With the advent of advanced techniques-such as proton spectroscopy, diffusion-weighted imaging (DWI), diffusion tensor imaging (DTI), magnetization transfer imaging, susceptibility-weighted imaging, perfusion-weighted imaging, T2/T2* relaxometry (quantification of iron overload), neuromelanin-sensitive MRI, and functional MRI-the role of MRI in the early diagnosis of Parkinson's disease has expanded, as has its role in the differential diagnosis between Parkinson's disease and atypical parkinsonism^(7-9)^. In addition, these advanced techniques correlate with scales of Parkinson's disease severity, also gaining importance in the staging and prognosis, as well as informing decisions regarding practice^(10,11)^.

### Conventional MRI

**Figure 1:**
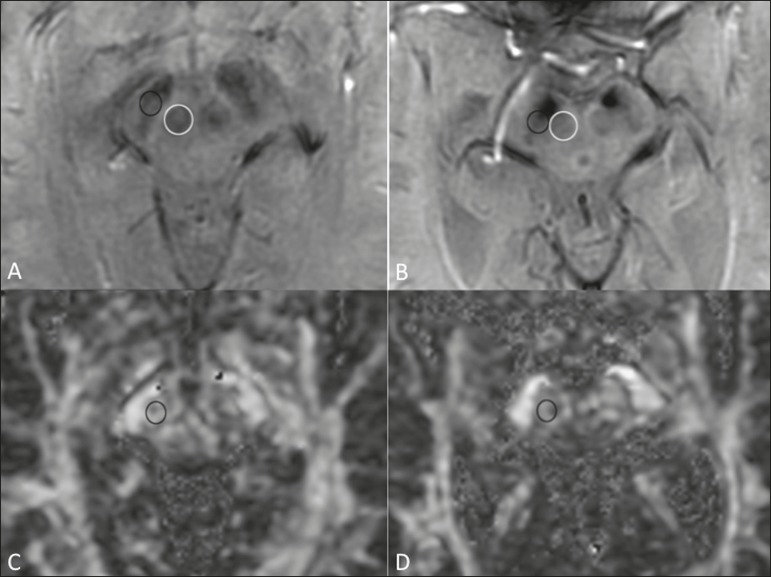
Findings on conventional MRI and DTI in Parkinson's disease. On T2-weighted sequences and susceptibility-weighted imaging, the normal substantia nigra presents as a band of hypointensity between the cerebral peduncle and the mesencephalic tegmentum. In a 66-year-old patient without Parkinson's disease (**A**), an axial slice of the midbrain on susceptibility-weighted imaging shows a well-delineated substantia nigra (black circle), of normal thickness, showing well-defined cleavage with the red nucleus (white circle). In a 68-year-old patient with Parkinson's disease (**B**), an axial slice of the midbrain on susceptibility-weighted imaging shows a poorly delineated, thin substantia nigra and poorly defined cleavage of the substantia nigra with the red nucleus. The reduced volume of the substantia nigra in the patient with Parkinson's disease is attributable to iron deposition. The regions of interest in the substantia nigra in patients **A** and **B** (black circles) were copied and pasted onto the FA map (**C** and **D**, respectively), which shows that the FA value was lower in the patient with Parkinson's disease than in the patient without (0.425 vs. 0.581), indicating a loss of neuronal integrity. The contralateral findings were comparable between the two patients (images not shown).

The results of conventional MRI scans with T1-weighted, T2-weighted, proton density-weighted, or fluid-attenuated inversion recovery sequences are usually normal in Parkinson's disease. Thinning of the substantia nigra pars compacta and diffuse cortical atrophy can occur in patients with Parkinson's disease (Figure 1), although those changes are typically seen only in the later stages of the disease^(12)^.

**Figure 2:**
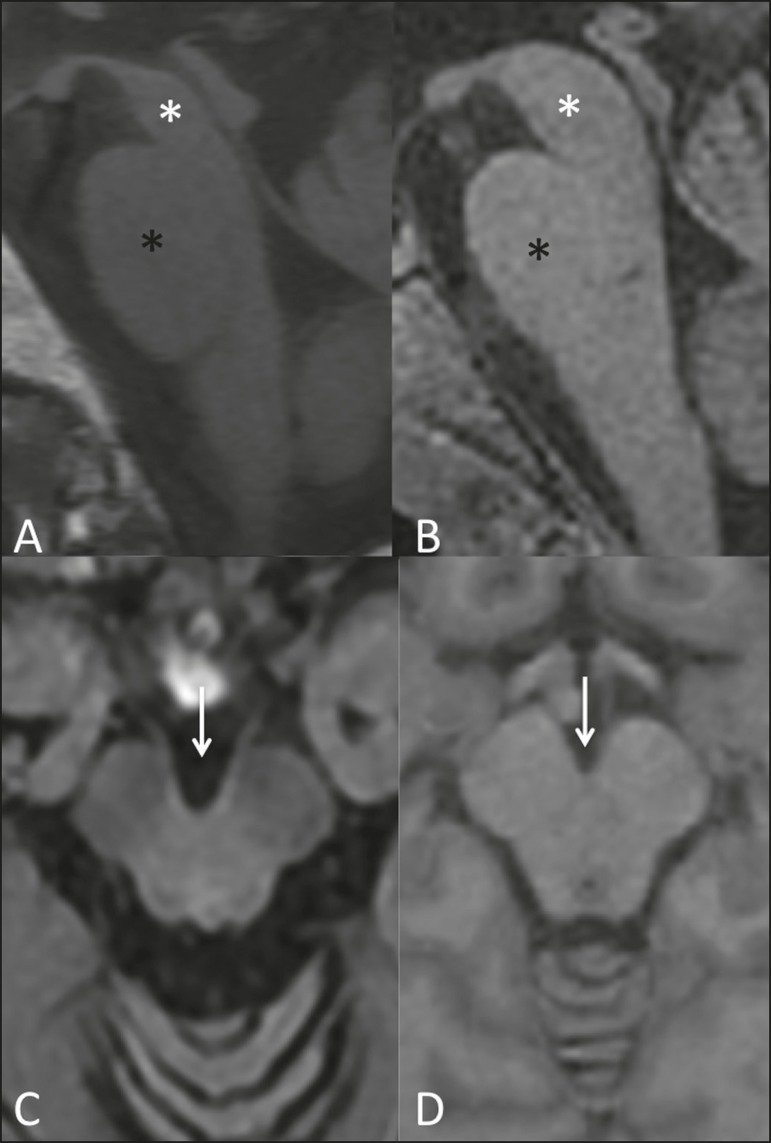
Conventional MRI findings in progressive supranuclear palsy. In a sagittal T1-weighted sequence, the hummingbird sign can be seen (**A**), due to the volumetric reduction of the midbrain (asterisk) in relation to the pons (black asterisk), unlike what is seen in the normal brainstem (**B**). In the axial plane, the Mickey Mouse sign (**C**) occurs due to widening of the interpeduncular cistern (white arrow) and retraction of the dorsolateral regions of the midbrain, none of which were observed in the control patient (**D**).

**Figure 3:**
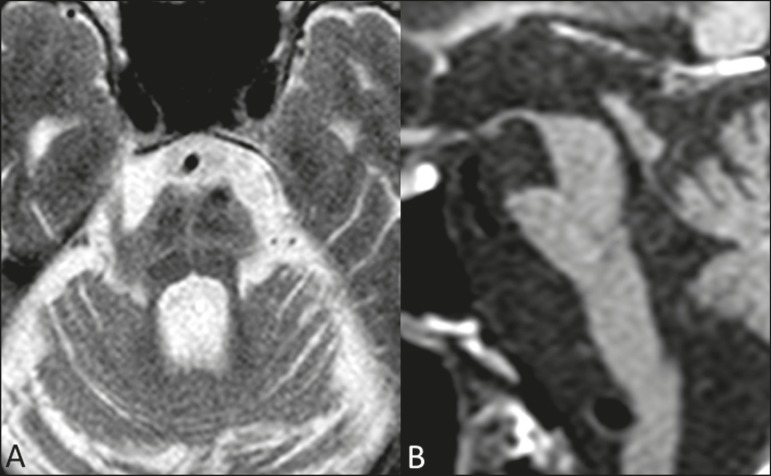
Morphological and signal changes on a conventional MRI scan of a patient with multiple system atrophy. The hot cross bun sign appears as two perpendicular lines in the pons, which present a hyperintense signal in an axial T2-weighted sequence, together with accentuation of the basal cisterns, cerebellar sulci, and fourth ventricle (**A**). A sagittal T1-weighted sequence (**B**) shows the volume reduction of the pons relative to the midbrain (compare with the normal brainstem shown in Figure 1B).

Conventional MRI can reveal major structural alterations that can facilitate the differential diagnosis between Parkinson's disease and atypical parkinsonism. In progressive supranuclear palsy, the hummingbird (or penguin) sign in the sagittal plane and the Mickey Mouse sign in the axial plane reflect the volumetric reduction of the midbrain^(13)^ , as depicted in Figure 2. In multiple system atrophy, a dorsolateral area of high signal intensity in the putamen can be seen in patients with a predominance of parkinsonian symptoms, whereas the hot cross bun sign is commonly seen in patients with predominant cerebellar symptoms in the advanced stages^(13)^, as shown in Figure 3. In addition to the qualitative visual evaluation, conventional MRI allows quantitative measurements to evaluate the volumetric reduction of the midbrain and superior cerebellar peduncles in progressive supranuclear palsy, as well as that of the pons, middle cerebellar peduncles, and cerebellar hemispheres in multiple system atrophy, thus increasing the diagnostic accuracy of the method^(14,15)^. Findings on conventional MRI play a less specific role in the diagnosis of corticobasal degeneration, because asymmetric atrophy of the parietal lobes and thinning of the corpus callosum have low specificity, although they can facilitate the diagnosis when accompanied by neurological signs^(16)^.

In a recent study of patients with Parkinson's disease or atypical parkinsonism, Massey et al.^(13)^, correlated the results of conventional MRI with neuropathological findings and showed that some MRI signs have high specificity. However, the authors stated that the low sensitivity of those signs, together with autopsy findings, underscores the need to develop imaging techniques that can detect microstructural changes in the absence of regional atrophy^(13)^.

### DWI and DTI

In recent years, DWI and DTI have emerged as promising tools for the identification and quantification of microstructural changes in regions of the brain that had hitherto appeared normal on conventional MRI^(17)^. These techniques provide a unique window for assessing changes in neuronal connectivity, allowing quantitative measurements of the integrity of cell nuclei and neuronal tracts^(18)^.

DWI quantifies the motion of water molecules by applying a diffusion sensitizing gradient in three orthogonal planes between two radiofrequency pulses^(17)^. The diffusivity of water in each plane is obtained by calculating the apparent diffusion coefficient (ADC), allowing the evaluation of the average diffusion coefficient of each tissue^(17)^. The random motion of water molecules is restricted by the normal architecture of the glial tissue and the neuronal tracts, a condition known as anisotropy^(17)^. The degree of anisotropy can be quantified by applying a diffusion sensitizing gradient in at least six directions (DTI), which allows the fractional anisotropy (FA) to be calculated^(17)^.

At the cellular level, anisotropy results from the presence of obstacles to water diffusivity due to the orientation of structures such as membranes, myelin, longitudinal filaments, and the cytoskeleton^(17,19)^. Changes in the ADC and FA can reflect axonal or myelin damage, which are common pathophysiological changes seen in neurodegenerative diseases^(9,19)^.

The quantitative analysis of DTI involves the manual placement of regions of interest (Figure 1) or automatic voxel-based methods, the two methods allowing similar assessments of the integrity of the brain microstructure^(20,21)^.

In view of the above, which indicates that some doubts remain, we decided to carry out a search of the current literature. Our intention was to update the knowledge about the role of MRI in the differential diagnosis between Parkinson's disease and atypical parkinsonism, with an emphasis on the diffusion technique.

## MATERIALS AND METHODS

We searched the Medline, PubMed, and SciELO databases, using descriptors in Portuguese and English. The Portuguese-language search terms were as follows: *doença de Parkinson; parkinsonismo; síndrome parkinsoniana; paralisia supranuclear progressiva; atrofia de múltiplos sistemas; degeneração corticobasal; imagem por ressonância magnética*; and *difusão por ressonância magnética*. The search terms in English were as follows: Parkinson disease; parkinsonism; parkinsonian syndrome; progressive supranuclear paralysis; multiple system atrophy; corticobasal degeneration; magnetic resonance imaging; and diffusion MRI. We limited our search to studies relevant to the study topic and published between February 2006 and February 2016.

In general, we selected studies related to Parkinson's disease, atypical parkinsonism, multiple system atrophy, progressive supranuclear palsy, corticobasal degeneration, MRI, DWI, and DTI. Although we focused on studies published more recently (within the last five years), we included some older articles that we considered to be of great relevance. Studies with poorly defined methodology or results were excluded, as were those in which the findings were inconclusive, those that were considered irrelevant, and those whose focus was not on the proposed theme.

## RESULTS

Of a total of 172 articles found, 7 were excluded because they were not published in Portuguese or English, and 125 were subsequently excluded because they did not meet the study criteria. Among the remaining 40 articles, we highlighted original and review articles that dealt with the role of DWI and DTI in Parkinson's disease and in the differential diagnosis with atypical parkinsonism.

### DIFFUSION IN PARKINSON'S DISEASE

It is estimated that, by the time a clinical diagnosis of Parkinson's disease is made, there has already been a loss of 50-70% of the dopaminergic neurons of the substantia nigra pars compacta^(22)^. Studies have shown that it is possible to detect changes in the orientation of water diffusivity (FA) within the substantia nigra in patients with Parkinson's disease (Figure 1), even in the early stages of the disease^(10,11,21-23)^.

Vaillancourt et al.^(22)^ found that the FA was reduced in the substantia nigra of untreated patients in the early stage of Parkinson's disease, the reduction being greater in its caudal portion than in its rostral portion. The authors also found that this distinction showed 100% accuracy in differentiating between such patients and healthy controls^(22)^.

In a recent systematic review and meta-analysis, Cochrane et al.^(8)^ evaluated nine studies that analyzed regions of interest of the substantia nigra, collectively involving 193 patients with Parkinson's disease and 195 control patients, and demonstrated a statistically significant reduction in FA among patients with Parkinson's disease. Among those studies, no changes in the ADC were detected, indicating a slight loss of microstructural integrity without gross tissue loss. Although reduced FA in the substantia nigra was consistently demonstrated in that meta-analysis^(8)^, it was not possible to draw reliable conclusions about the diagnostic accuracy of the distinction, making it difficult to characterize it as a viable biomarker of disease. Studies using automatic voxel-based methods have also demonstrated reduced FA in the substantia nigra, although they are scarcer in the literature and have used various methods of image post-processing, which impedes comparisons across studies and decreases the reproducibility of the findings^(21,24,25)^.

Changes in water diffusivity can be found in other regions of the brain in individuals with Parkinson's disease, even in its early stages, when there is as yet no significant cortical atrophy^(26)^. It has been reported that the FA is lower in the motor, premotor, and supplemental motor cortices of patients with Parkinson's disease than in those of control patients, probably due to degeneration in the corticostriatal and thalamocortical projections in the former^(21)^. It has also been demonstrated that patients with Parkinson's disease show increased FA in the post-central gyrus, in the projection from the somatosensory cortex, which correlates quite well with the severity of the disease and has been interpreted as a possible compensatory mechanism of the brain in response to the loss of motor control^(21)^.

Gattellaro et al.^(18)^ compared 10 patients with earlystage Parkinson's disease and 10 control patients. The authors reported that the patients with Parkinson's disease showed a lower FA in the genu of the corpus callosum and in the superior longitudinal fasciculus, whereas the corticospinal tract showed no alterations. Impaired water diffusivity in the genu of the corpus callosum can indicate degeneration of the interhemispheric axonal connections among frontal areas. Degeneration of specific areas of the frontal lobes was also well demonstrated in a study conducted by Kendi et al.^(27)^, who compared DTI and volumetric findings in patients with Parkinson's disease and controls, reporting neuronal degeneration even in patients without volumetric reduction of the frontal lobes.

Jiang et al.^(11)^ evaluated 31 patients with Parkinson's disease and 34 age- and gender-matched controls. The authors observed that the FA in the substantia nigra correlated with the stage of the disease according to the Hoehn and Yahr staging scale, whereas the FA in the frontal white matter correlated with the activities of daily living disease severity score, corroborating the hypothesis that DTI can play a role in the staging of the disease, as well as in the prognosis and decision-making regarding its management.

Changes in the diffusivity of the striatum and cerebellar hemispheres in patients with Parkinson's disease have also been reported and could represent potential areas of study to improve knowledge about the disease, especially regarding specific motor symptoms^(28,29)^.

DTI has also been explored in order to clarify the non-motor symptoms of Parkinson's disease, as well as to identify its anatomical and functional substrates.

Subtle cognitive deficits can occur in early-stage Parkinson's patients, especially in those who are elderly, although dementia typically occurs in patients who have reached the more advanced stages and is associated with an accelerated increase in morbidity and mortality^(30,31)^. Studies have demonstrated a reduction of the FA in the cingulate gyrus of patients with Parkinson's disease and cognitive alterations, suggesting that these neuronal fibers play an important role in the dementia associated with Parkinson's disease^(30-33)^. Other neuronal fibers that can be associated with dementia in Parkinson's disease are those of the corpus callosum, hippocampus, superior longitudinal fasciculus, inferior longitudinal fasciculus, inferior fronto-occipital fasciculus, and uncinate fasciculus^(32-36)^.

Depression occurs in approximately 40% of patients with Parkinson's disease and therefore has also been studied with DTI^(37)^. It has been demonstrated that patients with Parkinson's disease and depression show a reduction in the FA in the mid-dorsal portions of the thalamus, anterior margin of the cingulate gyrus, and white matter of the frontal lobes, which might be related to depressive symptoms and perhaps to the degree of impairment^(11,31,37)^.

Another example of a non-motor symptom in Parkinson's disease is olfactory dysfunction, which occurs in most patients, even before the onset of motor symptoms^(33,34)^. Alterations in diffusivity in the olfactory bulbs and the white matter adjacent to the primary olfactory cortex and the gyrus rectus have been reported; such alterations can be identified through specific studies using olfactory tests together with the clinical evaluation in the premotor phase of Parkinson's disease^(38-41)^.

A recent study showed that the quantification of non-Gaussian water diffusion (kurtosis) has been used in the evaluation of microstructural damage in neurodegenerative diseases, showing promise for use in Parkinson's disease in relation to conventional DTI analyses^(42)^.

### DIFFUSION IN THE DIFFERENTIAL DIAGNOSIS WITH ATYPICAL PARKINSONISM

Because DTI can assess neuronal integrity in different regions of the brain, it has emerged in the last decade as a potential tool for making the differential diagnosis between Parkinson's disease and parkinsonian syndromes.

Various studies have demonstrated changes in water diffusivity in the putamen, measured by region of interest, in patients with multiple system atrophy and a predominance of parkinsonian symptoms, allowing the differentiation with Parkinson's disease even in its early stages, when there is as yet no volumetric reduction or signal change on conventional MRI^(43-48)^. In a study conducted by Seppi et al.^(46)^, the increase in ADC was much more pronounced in the posterior portion of the putamen than in its anterior portion, which could be an early biomarker of the disease. Abnormalities of the putamen have also been reported in patients with progressive supranuclear palsy, which can reduce their specificity as an isolated finding in the diagnosis of multiple system atrophy^(49)^.

In patients with multiple system atrophy, reduced FA and an increased ADC are also typically found in the pons, middle cerebellar peduncles, and cerebellar hemispheres^(43,50-52)^. Ito et al.^(43)^ found that such changes in the putamen, pons, and cerebellar hemispheres had high accuracy in the differentiation between multiple system atrophy and Parkinson's disease, even in patients without classic signs on conventional MRI, and the reduction in FA in the pons had 70% sensitivity and 100% specificity.

Measurements of FA could show even greater accuracy in the differentiation between Parkinson's disease and multiple system atrophy if added to conventional MRI measurements, as demonstrated in a study conducted by Nair et al.^(53)^, who reported that the combination has 92% sensitivity and 96% specificity.

Patients with progressive supranuclear palsy also present changes in water diffusivity in specific regions of the brain. Studies have shown that the superior cerebellar peduncles are the regions that have the best reliability in the differential diagnosis between Parkinson's disease and progressive supranuclear palsy^(16,49,52)^. Nicoletti et al.^(49)^ demonstrated that elevated ADC in the superior cerebellar peduncles had 100% accuracy in differentiating patients with progressive supranuclear palsy from those with Parkinson's disease and from control patients. The authors reported that the finding also had 96.4% sensitivity and 93.3% specificity in differentiating between progressive supranuclear palsy and multiple system atrophy.

Patients with progressive supranuclear palsy can also present changes in diffusivity in the midbrain, caudate nucleus, and globus pallidus, which together can facilitate the differentiation with Parkinson's disease and multiple system atrophy^(47,49,50,54)^.

Due to its low prevalence, corticobasal degeneration has rarely been evaluated in diffusion MRI studies. Asymmetry of the motor symptoms, which is typical of the disease, correlates with reduced ADC in the cerebral hemisphere contralateral to the most affected side, as described by Rizzo et al.^(16)^. Boelmans et al.^(55)^ also reported alterations in the contralateral hemisphere, demonstrating an increased ADC and reduced FA in the corticospinal tract and corpus callosum of patients with corticobasal degeneration compared with those of control patients. Erbetta et al.^(56)^ reported an increased ADC in the contralateral motor thalamus, precentral gyrus, and postcentral gyrus of patients with corticobasal degeneration, demonstrating that it facilitates the differential diagnosis with progressive supranuclear palsy.

Recently, a task force has recommended the use of conventional diffusion-associated MRI in the differential diagnosis among Parkinson's disease, multiple system atrophy, and progressive supranuclear palsy, characterizing a Grade A recommendation^(57)^. Table 1 summarizes the findings on conventional and diffusion MRI in Parkinson's disease and atypical parkinsonism.

**Table 1:** Findings on conventional and diffusion MRI in Parkinson's disease and atypical parkinsonism.

	Conventional MRI			Diffusion-weighted MRI	
	Findings	Considerations		Findings	Considerations
PD	- Thinning of the substantia nigra pars compacta, best visualized in axial T2-weighted sequences and susceptibility-weighted imaging^(12)^	- Quite high specificity, although quite low sensitivity, found only in the more advanced stages^(12)^		- Reduced FA in the substantia nigra, more evident in the caudal portion^(8,10,11,21-25)^	- High specificity and sensitivity, even in the early stages of the disease, data more established in the literature^(8,10,11,21-25)^
				- Reduced FA in the frontal lobes (motor, premotor and supplemental motor cortices)^(18,21,27)^	- Low specificity and sensitivity^(18,21,27)^
				- Reduced FA in the striatum and cerebellar hemispheres^(28,29)^	- Rarely studied but could be associated with specific signs of the disease^(28,29)^
	- Diffuse cortical atrophy, mainly in the frontal lobes^(12)^	- Low sensitivity and specificity, occurs in the terminal phase^(12)^		- Reduced FA in the genu of the corpus callosum, cingulate gyrus, hip-pocampus, superior/inferior longitudinal fasciculi and inferior/uncinate fronto-occipital fasciculi^(30-36)^	- Appears to be related to dementia in PD but warrants further study^(30-36)^
				- Reduced FA in the dorsomedial thalamus and anterior cingulate gyrus^(11,31,37)^	- Could be related to depression, although it has not been extensively studied^(11,31,37)^
				- Reduced FA in the olfactory bulbs, white matter adjacent to the primary olfactory cortex, and gyrus rectus^(38,40,41)^	- Can be present in individuals with altered olfactory tests, with the potential to identify patients in the premotor phase of PD^(38,40,41)^
MSA	- Hot cross bun sign in axial T2-weighted sequences, with atrophy of the pons^(13-15)^	- All have good specificity but can be seen in other conditions^(13-15)^		- Reduced FA and increased ADC in the putamen^(43-48)^	- High sensitivity and specificity, especially in MSA-P, but can also occur in PSP^(43-48)^
	- Hyperintense signal in axial T2-weighted sequences, with atrophy of the putamen^(13-15)^	- Moderate sensitivity, because they are not normally seen in the early stages of the disease^(13-15)^		- Reduced FA and increased ADC in the pons, cerebellum, and middle cerebellar peduncles^(43,50-52)^	- High sensitivity and specificity, especially in MSA-C. In conjunction with changes in the putamen, they increase the specificity consider-ably^43,50-52)^
	- Atrophy of the cerebellar hemispheres and the middle cerebellar peduncles^(13-15)^				- Diffusion measures, taken together with conventional MRI measures, present high accuracy in the differential diagnosis with PD^(53)^
PSP	- Hummingbird or penguin sign in the sagittal plane^(13-15)^	- All have very high specificity, al-though they might not be seen in the early stages^(13-15)^		- Reduced FA in the midbrain and superior cerebellar peduncles^(16,49,52)^	- High sensitivity and specificity^(16,49,52)^
	- Mickey Mouse sign in the axial plane^(13-15)^			- Reduced FA in the globus pallidus and caudate nucleus^(47,49,50,54)^	- Can facilitate the differential diag-nosis but have not been widely studied^(47,49,50,54)^
	- Atrophy of midbrain and superior cerebellar peduncles^(13-15)^				
CBD	- Asymmetric atrophy of the parietal lobes^(16)^	- Low specificity and sensitivity^(16)^		- Reduced FA and increased ADC in the corpus callosum and frontal lobes, mainly in the corticospinal tract contralateral to the symptomatic side^(16,55)^	- Low sensitivity and specificity but can be seen in the early stages, even in the absence of atrophy^(16,55)^
	- Thinning of the corpus callosum^(16)^			- Increased ADC in the motor thala-mus, precentral gyrus, and postcentral gyrus^(56)^	- Rarely studied but can facilitate the differential diagnosis with PSP, especially if asymmetric^(56)^

PD, Parkinson's disease; MSA, multiple system atrophy; PSP, progressive supranuclear palsy; CBD, corticobasal degeneration; FA, fractional anisotropy; ADC, apparent diffusion coefficient; MSA-P, MSA with a predominance of parkinsonian symptoms; MSA-C, MSA with a predominance of cerebellar symptoms.

## CONCLUSION

Because of its ability to detect changes in the microstructure of the brain damage, the use of diffusion MRI in patients with neurodegenerative diseases has been extensively studied in recent years. With technological development, the role that diffusion MRI plays in the differential diagnosis between Parkinson's disease and atypical parkinsonism has expanded, thus increasing our knowledge of these diseases, as well as facilitating the identification of their anatomical and functional substrates.

Despite the promising findings, there is a need for further studies, involving larger cohorts and longitudinal designs, in order to increase the rates of early diagnosis of Parkinson's disease, as well as to improve staging, prognostic evaluation, and treatment planning among patients with the disease.
